# “Cerberus” T Cells: A Glucocorticoid-Resistant, Multi-Pathogen Specific T Cell Product to Fight Infections in Severely Immunocompromised Patients

**DOI:** 10.3389/fimmu.2020.608701

**Published:** 2021-01-18

**Authors:** Kiriakos Koukoulias, Penelope-Georgia Papayanni, Aphrodite Georgakopoulou, Maria Alvanou, Stamatia Laidou, Anastasios Kouimtzidis, Chrysoula Pantazi, Glykeria Gkoliou, Timoleon-Achilleas Vyzantiadis, Alexandros Spyridonidis, Antonios Makris, Anastasia Chatzidimitriou, Nikoletta Psatha, Achilles Anagnostopoulos, Evangelia Yannaki, Anastasia Papadopoulou

**Affiliations:** ^1^ Hematology Department, Hematopoietic Cell Transplantation Unit, Gene and Cell Therapy Center, “George Papanikolaou” Hospital, Thessaloniki, Greece; ^2^ Department of Genetics, Development and Molecular Biology, School of Biology, Aristotle University of Thessaloniki, Thessaloniki, Greece; ^3^ Department of Internal Medicine, BMT Unit, University of Patras, Patras, Greece; ^4^ Institute of Applied Biosciences (INAB), Centre for Research and Technology Hellas (CERTH), Thessaloniki, Greece; ^5^ First Department of Microbiology, Medical School, Aristotle University of Thessaloniki, Thessaloniki, Greece; ^6^ Altius Institute for Biomedical Sciences, Seattle, WA, United States; ^7^ Department of Medicine, University of Washington, Seattle, WA, United States

**Keywords:** cytomegalovirus, Epstein-Barr virus, adenovirus, BK virus, *Aspergillus fumigatus*, T cell therapy

## Abstract

Adoptive immunotherapy (AI) with pathogen-specific T cells is a promising alternative to pharmacotherapy for the treatment of opportunistic infections after allogeneic hematopoietic cell transplantation or solid organ transplantation. However, clinical implementation of AI is limited to patients not receiving high-dose steroids, a prerequisite for optimal T-cell function, practically excluding the most susceptible to infections patients from the benefits of AI. To address this issue, we here rapidly generated, clinical doses of a steroid-resistant T-cell product, simultaneously targeting four viruses (adenovirus, cytomegalovirus, Epstein Barr virus, and BK virus) and the fungus *Aspergillus fumigatus*, by genetic disruption of the glucocorticoid receptor (GR) gene using CRISPR/CAS9 ribonucleoprotein delivery. The product, “Cerberus” T cells (Cb-STs), was called after the monstrous three-headed dog of Greek mythology, due to its triple potential; specificity against viruses, specificity against fungi and resistance to glucocorticoids. Following efficient on-target GR disruption and minimal off-target editing, the generated Cb-STs maintained the characteristics of pentavalent-STs, their unedited counterparts, including polyclonality, memory immunophenotype, specificity, and cytotoxicity while they presented functional resistance to dexamethasone. Cb-STs may become a powerful, one-time treatment for severely immunosuppressed patients under glucocorticoids who suffer from multiple, life-threatening infections post-transplant, and for whom therapeutic choices are limited.

## Introduction

The outcome of allogeneic hematopoietic cell transplantation (allo-HCT) and solid organ transplantation (SOT) is severely impaired by the development of opportunistic infections from viruses and/or fungi (1–3). Infections from cytomegalovirus (CMV), Epstein-Barr virus (EBV), polyoma virus type I (BKV), adenovirus (AdV), and *Aspergillus fumigatus* (AF) affect the majority of transplanted patients, especially those under intense immunosuppression with high-dose glucocorticoids to control the immunological complications of transplantation (3, 4).

Today’s standard treatment of infections with pharmacological agents often fails, while it may lead to toxicity/intolerance or the outgrowth of drug-resistant strains (5–10). Despite the introduction of preemptive antiviral therapy in routine post-transplant care, infection-related mortality post allo-HCT remains at 12–27% (11). As opposed to drug treatment, adoptive immunotherapy (AI) with virus-specific T cells (VSTs) is a more natural way to fight pathogens (12–21), holding great promise as a novel cell therapy tool for the treatment of infections post-transplant.

Notwithstanding the significant clinical progress with VSTs, there are certain limitations yet to be overcome towards a broader use of AI. First, despite the broadening of the target repertoire of VSTs with the transition from single to multivalent VSTs (20, 22), fungi have not yet been targeted by a composite T-cell product. In fact, antifungal AI has not reached by any means, the success of viral immunotherapy (23). Second, immunosuppressive drugs significantly impair T-cell functionality (24–28), confining the use of antigen-specific T cells only to patients in whom immunosuppression has been tapered or withdrawn. The latter, creates the paradox of precluding from the potential benefits of AI, the most vulnerable to life-threatening infections patients; those receiving high-dose glucocorticoids, the first-line treatment of graft-versus-host disease (GvHD) post HCT or rejection post SOT.

To overcome current limitations of AI with Ag-specific T cells (23), we here generated T-cell products with multi-pathogen specificity and concurrent glucocorticoid-resistance. These cells simultaneously target four viruses (CMV, EBV, AdV, BKV) and the fungus AF, while being resistant to glucocorticoids, *via* clustered regularly interspaced short palindromic repeats/Cas9 (CRISPR/Cas9)-mediated disruption of the glucocorticoid receptor (GR). These “multi-talented” T cells exhibit a triple potential of specificity against viruses, specificity against fungi and resistance to glucocorticoids that inspired us to call them, “Cerberus” T cells (Cb-STs), from the three-headed dog of Greek mythology. Like “Cerberus” who guarded the gates of the underworld, Cb-STs may serve as a powerful guard system against multiple pathogens for transplanted patients, even under the unfavorable condition of intense immunosuppression.

## Materials and Methods

### Healthy Donors

The study was approved by the Institutional Review Board of the George Papanikolaou hospital. Under signed informed consent, peripheral blood from healthy volunteers was obtained for the generation of antigen-specific T cells.

### Lentiviral Plasmid Construction and Viruses

LentiCRISPR v2 was a gift from Feng Zhang (Addgene plasmid # 52961) (29). DNA sequences of all gRNAs used for GR gene knockout are listed as 5′ to 3′ sequences in **Table S1**. The sequence of gRNAs used for gene knockout were designed using the Vector NTI software (Thermo Fisher).

Cloning of gRNAs into LentiCRISPR v2 was performed according to Sanjana et al. and Shalem et al. (29, 30). The lentiCRISPR vector was digested and dephosporylated with FastDigest BsmBI and FastAP (Thermo Fisher) at 37°C for 30 min and gel-purified on a 1% agarose gel using DNA Gel Extraction kit (Bioline), according to the manufacturer’s recommendations. Oligonucleotides for the sgRNA guide sequence (Invitrogen) were phosphorylated using polynucleotide kinase (NEB) at 37°C for 30 min and then annealed by heating to 95°C for 5 min and cooling to 25°C at 5°C/min. Using Quick ligase (NEB), annealed oligos were ligated into gel purified vectors (Qiagen) at RT for 10 min. The cloned constructs were then transformed into Stbl3 chemically competent *E. coli* (invitrogen) according to the manufacturer’s protocol. Cloned transfer plasmids were amplified using a maxi-prep kit (Macherey-nagel). Diagnostic digest was performed for confirming the positive clones. The colonies with positive insertion were confirmed by analyzing the resulting fragments by gel electrophoresis and colony PCR.

The lentiviral vector was produced by 293T cells transient cotransfection with transfer vector, gag–pol construct, and VSV-G envelope construct (kindly provided by Emery DW) according to established protocol (31), and was harvested and filtered before used for transduction.

### Transduction and Selection of Transduced T2 Cells

For each viral construct, 2x10^5^ T2 cells (ATCC) were transduced in suspension with viral supernatant. Every 2–3 days onwards, media was replenished with fresh medium containing 0.25 μg/ml puromycin (Invivogen).

### Vector Copy Number (VCN) Analysis

Real-time quantitative PCR was performed in ABI 7500 (Applied Biosystems) using the following primers and probes: Gag F: 5’-GGA-GCT-AGA-ACG-ATT-CGC-AGT-TA-3’, Gag R: 5’-GGT-TGT-AGC-TGT-CCC-AGT-ATT-TGTC-3’, Gag Probe: 5’-FAM-ACA-GCC-TTC-TGA-TGT-TTC-TAA-CAG-GCC-AGG-TAMRA-3’, hAlb F: 5’-TGA-AAC-ATA-CGT-TCC-CAA-AGA-GTTT-3’, hAlb R: 5’-CTC-TCC-TTC-TCA-GAA-AGT-GTG-CAT-AT-3’, hAlb Probe: 5’-VIC-TGC-TGA-AAC-ATT-CAC-CTT-CCA-TGC-AGA-TAMRA-3’. Each DNA sample was run in triplicate in 25 μl reaction volume using Taqman Universal PCR Master Mix (Applied Biosystems). Thermal cycling was started for 2 min at 50°C, followed by 10 min at 95°C, 40 thermal cycles of 15 s at 95°C and 1 min at 60°C. Vector copy number/cell was calculated by normalizing to the endogenous ALB gene using the following formula: (quantity mean of GAG sequence/quantity mean of ALB sequence) ×2 (2-fold factor was used because DNA derived from the diploid cells).

### T7 Endonuclease I Assay

Genomic DNA was isolated using a QIAamp DNA Mini Kit (Qiagen). PCR to amplify targeted locus was performed for 30–35 cycles using Q5 High-Fidelity DNA Polymerase and the respective set of primers. PCR products was reannealed in NEB 2 buffer and were treated with 10 Units of T7 Endonuclease I (NEB) at 37°C for 20 min. Reactions were stopped by the addition of EDTA and were electrophorated in 1.5% agarose gel stained with SYBR Green I nucleic acid gel stain (ThermoFisher). Band intensity was analyzed using ImageJ software (NIH). The gene modification levels were calculated using the following formula: 

% cleavage={1−sqrt[parental band/(parental band+cleaved bands)]×100%}

### Cas9 Ribonucleoproteins (RNPs) Preparations and Electroporation

sgRNA was synthesized and purified using EnGen sgRNA Synthesis Kit and Monarch RNA Cleanup Kit, respectively as per manufacturer’s instructions (New England Biolabs- NEB). The newly formed sgRNA (180 pmol for 1.5x10^7^ cells) was mixed with 60 pmol EnGen Cas9 NLS, *Streptococcus pyogenes* (NEB) and incubated at 25°C for 10min. Subsequently the fresh precomplexed RNP was added at the cells previously resuspended at nucleofector solution (human T cell nucleofector kit, LONZA) and were immediately electroporated using the AMAXA Nucleofector II (Program T-007, Lonza).

### Antigen-Specific T Cell Generation

Cb-STs and pentavalent-specific T cells (penta-STs) targeting four viruses (AdV, CMV, EBV, and BKV) and the fungus AF were generated by pulsing a total of 4.5x10^7^ peripheral blood mononuclear cells (PBMCs), deriving from 35 ml blood from normal EBV and/or CMV seropositive donors, to a mastermix of 0.5 μg/ml viral peptides [AdV: Hexon and penton; CMV: immediate early 1 (IE1) and pp65; EBV: Epstein–Barr nuclear antigen 1 (EBNA1), Latent Membrane Protein 2 (LMP2), BZLF1 and BKV: Large T, VP1] combined with 1 μg/ml AF peptides (Crf1, Gel1, SHMT) and culturing the cells in VST media [Advanced RPMI 1640 supplemented with 45% Click’s medium, 2 mM GlutaMAX, and 10% FBS] supplemented with interleukin 7 (IL-7) and IL-4 in G-Rex10 devices (Wilson Wolf Manufacturing Corporation), as previously described (20, 32). Three days after activation, 3x10^7^ cells were electroporated with RNPs as described above and subsequently cultured for 3–4 days, while the remaining unedited penta-STs served as control group (**Figure S1**). On day 6–8, cultures were either replenished with fresh VST media and cytokines or if they had reached a density >5x10^7^/G-Rex10 the cultures were split and fed.

### Phytohaemagglutinin (PHA) Blast Generation

PHA blasts were generated from PBMCs as previously described (32) by using PHA (1.5%) and maintaining the cells in VST media supplemented with IL2 (100 U/ml), replenished every 3 days.

### Flow Cytometry (FCM)

#### Cell Cycle Analysis

Cells were washed with PBS and resuspended in 1:10 PBS:70% pre-chilled ethanol and incubated for at least 2 h at -20°C. Subsequently cells were washed twice with PBS, resuspended at Propidium Iodide/RNAse solution (Immunostep) and analyzed by FCM.

#### Apoptosis Assay

Cell apoptosis was measured after staining with annexiv V and propidium iodide (exbio), as per manufacturer’s instructions. In brief, cells were washed with PBS and were incubated with annexiv V and propidium iodide for 15 min. After incubation, they were washed, resuspended in buffer, and analyzed by FCM.

#### GR Staining

The GR staining was performed with knockout validated anti-GR antibody (Abcam) and an intracellular staining kit (exbio) based on manufacturer’s recommendations with some modification. In particular, cells were washed with blocking buffer (PBS containing 0.5% BSA), fixed, permeabilized for 30 min and well-washed. Cells were subsequently stained with anti-GR for 30 min, well-washed, stained with a secondary goat anti-rabbit IgG (Abcam) for 30 min, well-washed and analyzed by FCM.

#### Immunophenotyping

T-cell products were stained with antibodies to human CD3, CD4, CD8, CD25, CD45RA, CD62L. T-cell subsets were defined as follows: naive; CD3^+^CD45RA^+^CD62L^+^, effector memory (Tem); CD3^+^CD45RA^−^CD62L^−^, central memory (Tcm); CD3^+^CD45RA^−^CD62L^+^ and terminally differentiated effector memory expressing CD45RA (TEMRA); CD3^+^CD45RA^+^CD62L^−^.

#### Proliferation

T-cell products were labeled with Carboxyfluorescein succinimidyl ester (CFSE) by incubation for 5 min at RT with 5μM CFSE in PBS containing 5% FBS. Cells were washed twice and after pulsing with PHA were cultured for 7 days in the presence and absence of different concentrations of dexamethasone (DEX) or Methylprednisolone before FCM analysis. Unpulsed cells served as negative control.

#### Cytotoxicity Assay

Cytotoxicity against antigen-pulsed and unpulsed PHA blasts was performed as previously described (33) with some modifications. In brief, autologous non-pulsed PHA blasts were labeled with a low concentration of CFSE (0.625 μM), while 2 h-antigen-pulsed autologous or unpulsed allogeneic PHA blasts were labelled with high concentration of CFSE (5 μM). Both CFSE-stained populations were mixed (1:1) and co-cultured with effector cells at various effector (E) to target (T) ratios. PHA blasts were maintained at 2x10^4^ cells in all conditions. Triplicates were performed for each condition. After 20 h-incubation, cells were stained with 7AAD in order to exclude dead cells. Using the ratio of CFSE^high^ and CFSE^low^ alive target cells without the presence of effector cells as baseline, the percentage of specific cytotoxicity was calculated based on the following equation:

$$\text{Cell lysis}(\%)=100-[100*\mathrm{Sample}(\text{CFS}{\text{E}}^{\text{high}}/\text{CFS}{\text{E}}^{\text{low}})/\text{Baseline}(\text{CFS}{\text{E}}^{\text{high}}/\text{CFS}{\text{E}}^{\mathrm{low}})]$$

All analyses were performed in a FACS Calibur device with the CellQuest Pro6 software (Becton Dickinson).

### Enzyme-Linked Immunospot (ELIspot) Assay

Antigen-specific T-cell products were pulsed with either their initial stimuli and the secretion of interferon-gamma (IFN-γ) or tumor-necrosis factor α (TNF-α) by the stimulated cells was measured by Elispot (Mabtech and Immunospot, respectively). Spot-forming cells (SFCs) were counted on Eli.Scan Elispot scanner (A.EL.VIS) using Eli.Analyse software V6.2.SFC. The specificity of cells was expressed as SFCs per input cell numbers. Response was considered positive if the total IFN-γ- or TNF-α–producing SFCs against each antigen tested, were ≥30 per 2×10^5^ input cells.

### Preparation of Fungal Lysates and Anti-Fungal Activity Testing

Aspergillus conidia were prepared from fresh, mature (2–5 days old) cultures of AF (A.T.C.C. 2004305) and the anti-fungal activity of T-cell products was determined on the basis of hyphal damage by the colorimetric assay with 2,3-bis[2-methoxy-4-nitro-5-sulfophenyl]2H-tetrazolium-5-carboxyanilide sodium salt (XTT; Thermo Fisher), as previously described (34). Water-soluble antigens were prepared from clinical specimen isolates of filamentous fungi (*Aspergillus Niger, Aspergillus Flavus, Fusarium solani, Fusarium oxysporum*) as previously described (34).

### ATG Treatment

To test whether Cb-STs are susceptible to complement-mediated cell lysis by ATG, 1x10^6^ cells/ml were cultured in VST medium supplemented with 50% active human serum in the presence and absence of ATG (100 μg/ml, Fresenius), as previously described (35). After incubation for 45 min, cells were stained with 7-AAD and the percentage of dead cells was measured by flow cytometry, while the complement-mediated cell lysis was calculated by the following formula: (% dead cells in ATG presence -% dead cells in medium without ATG)/(100- % dead cells in medium without ATG)×100.

### On-Target and Off-Target Data Analysis

Potential off-target sites were predicted using the Cas-OFFinder online tool (36). Genomic DNA was used for amplicon specific PCR using genome specific primers which flanked the expected on-target site for GR CRISPR/Cas9 target, and 7 (2 exonic and 5 intronic) in silico predicted potential offsite targets (named Off-site1-7; **Table S2**). The eight PCR products from each sample were pooled and 100 ng of the final pool were used as input for the library construction with the NEBNext Ultra II Library Prep kit (New England Biolabs, #E7645). Library quantification was performed with the KAPA SYBR FAST Universal qPCR kit (KAPA Biosystems, #4824) and the indexed libraries were paired-end sequenced on the NextSeq 500 Illumina platform. Fastq data were analyzed with Cas-analyzer (http://www.rgenome.net/cas-analyzer/) (37).

### Statistical Analysis

Results are expressed as mean ± standard error of the mean (SEM). Differences between data sets were analyzed using one-way analysis of variance (ANOVA) followed by Dunnett’s multiple comparisons test when comparing with the mean of a control column, or Tukey’s post-hoc test when comparing with the mean of every other column for multiple comparisons or a 2-tailed Student’s t-test for two group comparison. Cytotoxicity between treatment groups in the presence of DEX was analyzed by two-way ANOVA followed by Dunnett’s post-hoc test. P-values ≤0.05 were considered significant.

## Results

### On-Target CRISPR/Cas9-Mediated Inactivation of the GR

To knockout the GR, we designed and evaluated 8 sgRNAs targeting various domains of the GR gene, including exons 1, 2, 4, and 5, corresponding to N-terminal domain, DNA-binding domain and hinge region respectively, and named as e1, e2a, e2b, e2c, e2d, e4, e5a, e5b (**Table S1**).

**Figure 1:**
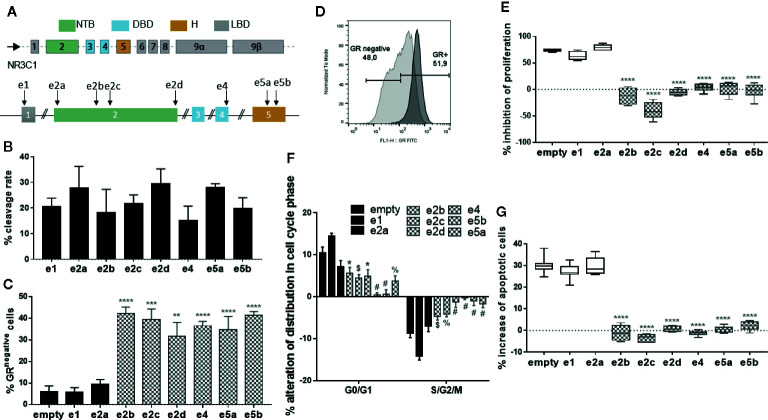
CRISPR/Cas9 mediated disruption of the GR in the T2 cell line and resistance of CRISPR/Cas9-edited T2 cells to glucocorticoids. **(A)** Schematic representation of the GR gene and different sgRNAs targeting exons 1,2, 4, and 5. NTB: N-terminal domain; DBD: DNA-binding domain; H: hinge region; LBD: ligand-binding domain. **(B)** Efficient on-target cleavage of the GR gene by all the gRNAs tested, as measured by T7 endonuclease I assay. Columns represent the mean ± SEM (n = 2–3). **(C)** Efficient on-target disruption of the GR protein by 6 (filled grey) of 8 sgRNAs tested, as shown by flow cytometry. Columns represent the mean ± SEM (n = 3–8). Differences between data sets were analyzed using one-way analysis of variance (ANOVA) followed by Dunnett’s post-hoc test. **p = 0.002 vs empty; ***p < 0.001 vs empty; ****p < 0.0001 vs empty. **(D)** Representative flow cytometry histogram showing the disruption of the GR protein in 48% of edited T2 cells (light grey), compared to the unedited cells (dark grey). **(E)** Box plots of inhibition of proliferation of edited and unedited (empty) T2 cells in the presence of dexamethasone (DEX), showing resistance of the proliferation of the T2 cells edited with 6 (filled grey) of 8 gRNAs. Inhibition is expressed relative to untreated (NO DEX) counterparts (n = 6–12). Differences between data sets were analyzed using one-way analysis of variance (ANOVA) followed by Dunnett’s post-hoc test. ****p < 0.0001 vs empty. **(F)** Alteration of distribution in cell cycle phase of edited and unedited (empty) T2 cells in the presence of DEX) showing resistance of the proliferation of the T2 cells edited with 6 (filled grey) of 8 gRNAs (n = 6–9). Inhibition is expressed relative to untreated (NO DEX) counterparts. Differences between data sets were analyzed using one-way analysis of variance (ANOVA) followed by Dunnett’s post-hoc test. *p < 0.05 vs empty; ^$^p < 0.01 vs empty; ^%^p < 0.001 vs empty; ^#^p < 0.0001 vs empty. **(G)** Box plots of increase of apoptosis of edited and unedited (empty) T2 cells in the presence of DEX, showing resistance of the apoptosis of the T2 cells edited with 6 (filled grey) of 8 gRNAs (n = 6–11). Increase is expressed relative to untreated (NO DEX) counterparts. Differences between data sets were analyzed using one-way analysis of variance (ANOVA) followed by Dunnett’s post-hoc test. ****p < 0.0001 vs empty.

The gRNAs were delivered in the T2 lymphoblastic cell line by lentiviral vectors encoding Cas9 and each of the 8 gRNAs targeting the GR. T2 cells were efficiently transduced, with a median of 0.8 vector copy number after puromycin selection (VCN; range 0.5–1; **Table S3**). T7 endonuclease I assay showed successful editing rate with up to 29.5% on-target introduction of insertions and deletions (indels) at the expected sites for all the gRNAs tested (**Figures 1A, B**, **Figure S2**). The efficient, on-target cleavage was not always translated to protein knock-out/down. Specifically, flow cytometry analysis revealed no effect in GR expression after targeting exon 1, which was not unexpected since it represents the 5’-untranslated region (5’-UTR) of the gene (38, 39). However, targeting the exon 2 proximally to methionine 1 (Met1), the first initiator codon resulting in the GR-A isoform, also failed to functionally disrupt the GR, indicating that translation might start from an alternative translational initiation site, such as Met27, resulting in the translational isoform GR-B, which is more transcriptionally active (39, 40) Targeting all the downstream loci in exons 2, 4, and 5 resulted in significant reductions in the percentage of cells expressing the GR, in approximately 32–42% of the cells (**Figures 1C, D**).

### Resistance of CRISPR/Cas9-Edited Cells to Glucocorticoids

To assess whether the CRISPR/Cas9-mediated mutations conferred resistance to glucocorticoids, puromycin-selected, transduced T2 cells were subsequently cultured in the presence or absence of high DEX concentration (10^-4^ M) for 7 days. Cells transduced with an “empty” viral vector expressing Cas9, but no gRNA, served as negative control. We observed that in the presence of DEX and relative to their untreated counterparts, the proliferation of empty-vector transduced T2 cells was strongly inhibited whereas T2 cells edited with 6 of 8 gRNAs (all except e1 and e2a), presented normal, uninhibited proliferation (**Figure S3**, **Figure 1E**).

Next, we examined the influence of DEX in cell cycle and the distribution of GR-edited cells across the different cell cycle phases over the empty-vector transduced cells. DEX treatment of control cells significantly increased the % of cells in G0/G1 phase and decreased the % of cells in S/G2/M phase over the GR-edited T2 cells (all but e1 and e2a), implying that GR-KO cells are still able to proliferate in the presence of DEX (**Figure 1F**, **Figure S4**). In addition, DEX induced apoptosis on empty-vector transduced T2 cells whereas edited cells (all but e1 and e2a) were protected from DEX’s apoptotic effects (**Figure 1G**), clearly suggesting functional DEX-resistance.

Among the 6 effective gRNAs in disrupting the GR and conferring resistance of edited cells to steroids, the gRNA e2b which presented the highest on-target and off-target specificity score (90, range 39–90) (**Table S1**) and conceivably the lower off-target activity, was selected for the disruption of GR and Cb-ST generation.

### Generation of Cb-STs

**Figure 2:**
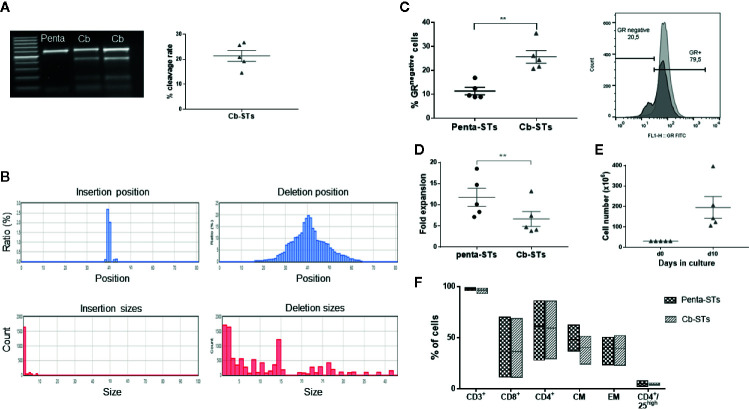
Generation and phenotypic characterization of Cb-STs. **(A)** Confirmation of the GR knockout at the gene level as measured by T7 endonuclease assay (left panel). The figure depicts the % cleavage rate (right panel). Each dot represents a single T-cell product (n = 5). **(B)** Representative on-target indels of a Cb-ST product using MiSeq analysis. **(C)** Confirmation of the GR knockout at the protein level as measured by flow cytometry. Each dot represents a single T-cell product (n = 5). Differences between data sets were analyzed using 2-tailed Student’s t-test. **p = 0.002. **(D)** Fold expansion of Cb-STs (grey triangles) and their unedited counterparts (penta-STs, black circles). Each dot represents a single T-cell product (n = 5). Differences between data sets were analyzed using 2-tailed Student’s t-test. **p = 0.002. **(E)** Absolute cell numbers of Cb-STs obtained after a 10-day culture in G-rex bioreactors. Each dot represents a single T-cell product (n = 5). **(F)** Immunophenotype of the generated Cb-STs (grey; n = 5) and their unedited counterparts (penta-STs, black; n = 5). Cb-STs, “Cerberus” T cells; Penta-STs, pentavalent-specific T cells; CM, central memory; EM, effector memory.

Towards increasing safety and simplifying Good manufacturing practice (GMP) production for future clinical trial, we proceeded to Cb-STs generation using an RNP complex for CRISPR/Cas9-sgRNA delivery by electroporation. In particular, pentavalent-specific T cells (penta-STs) targeting 4 viruses (AdV, CMV, EBV, and BKV) and the fungus AF were generated based on a previously optimized protocol for multivirus-specific or aspergillus-specific T cells (n = 5 different donors) and then edited using the optimal e2b sgRNA to generate Cb-STs (**Figure S1**) (20, 34). Unedited penta-STs from the same donor served as control group. At the end of the culture, the on-target editing of Cb-STs was evaluated at the gene level, showing an average of 21.4 ± 2.2% cleavage rate of the GR genomic sequence (**Figure 2A**). Deep-sequencing analysis (MiSeq) of the targeted exon 2 sequence confirmed the high specificity of the CRISPR/Cas9 on-target mutations, showing a high proportion of deletions, with insertions being on average only 17% of the total indels (**Figure 2B**), resulting in an average of 25.7 ± 2.6% GR^-^ cells (**Figure 2C**). Despite the reduced expansion rate of Cb-STs as compared to penta-STs (p = 0.002) due to the applied electroporation, we produced an average of 1.94 ± 0.5x10^8^ cells, starting from just 25–30ml of peripheral blood (**Figures 2D, E**).

**Figure 3:**
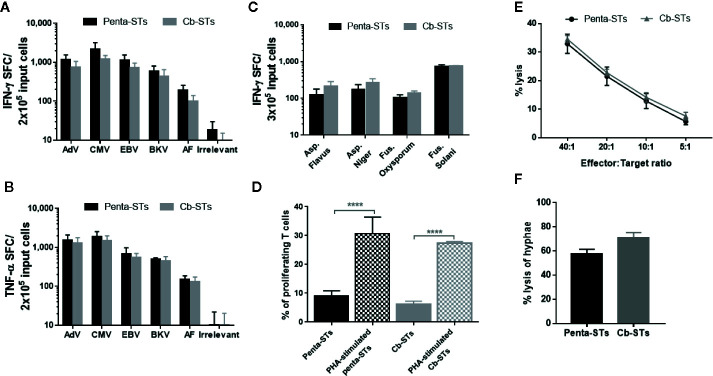
Functional characterization of Cb-STs. A–B) IFN-γ **(A)** and TNF-α **(B)** secretion of the generated Cb-STs (grey columns; n = 5) and their unedited counterparts (penta-STs; black columns; n = 5) upon stimulation with their initial stimuli. Columns represent the mean ± SEM. **(C)** Cross-reactivity of penta-STs (black columns; n = 3) and Cb-STs (grey columns; n = 4) by re-stimulation with various fungal lysates and measurement of IFN-γ producing cells by Elispot. Columns represent the mean ± SEM. **(D)** Proliferative capacity of the generated Cb-STs (grey columns; n = 2 in triplicates) and their unedited counterparts (penta-STs; black columns; n = 2 in triplicates) with or without restimulation with PHA. Columns represent the mean ± SEM. Differences between data sets were analyzed using one-way analysis of variance (ANOVA) followed by Tukey’s post-hoc test. ***p < 0.0001. **(E)** Cytotoxic activity of the generated Cb-STs (grey line; n = 5 in duplicates or triplicates) and their unedited counterparts (penta-STs; black line; n = 5 in duplicates or triplicates) against autologous, peptide-pulsed PHA blasts. **(F)** Antifungal activity of the generated Cb-STs (grey column; n = 2 in triplicates) and their unedited counterparts (penta-STs; black column; n = 2 in triplicates). Columns represent the mean ± SEM. Cb-STs, “Cerberus” T cells; Penta-STs, pentavalent-specific T cells; IFN-γ, interferon-γ; AdV, adenovirus; CMV, cytomegalovirus; EBV, Epstein Barr virus; BKV, BK virus; AF, *Aspergillus fumigatus*; TNF-α, tumor necrosis factor-α; PHA, Phytohaemagglutinin.

Importantly, Cb-STs maintained the penta-ST’s phenotype, containing both helper and effector cells and expressing memory markers with only a negligible number of CD4+/CD25^high^ regulatory T cells (**Figure 2F**). Similarly, after re-exposure of each T-cell product to its initial stimuli and IFN-γ and TNF-α secretion measurement by ELIspot assay, the functionality of Cb-STs was comparable to the corresponding specificity of Penta-STs, against all targeted pathogens (**Figures 3A, B**). As we have previously shown with Asp-STs (34) and multi-pathogen-specific T cells targeting AF (41, in press), Cb-STs displayed broad anti-fungal cross-immunity showing specificity also against other Aspergillus genera (Aspergillus Flavus, Aspergillus Niger), and fungi species (Fusarium; Oxysporum and Solani; **Figure 3C**). In addition, Cb-STs were capable of proliferating upon re-stimulation with PHA (**Figure 3D**), while inducing similar to penta-STs, strong and specific lysis of both autologous AdV-pulsed PHA blasts and AF hyphae (**Figures 3E, F**).

### In Vitro Resistance of Cb-STs to Glucocorticoids

**Figure 4:**
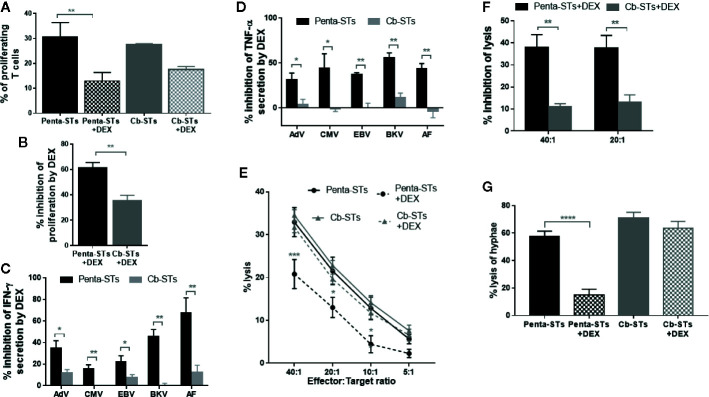
In vitro resistance of Cb-STs to glucocorticoids. **(A)** Proliferation of PHA-stimulated penta-STs (black columns; n = 2 in triplicates) and Cb-STs (grey columns; n = 2 in triplicates) in the presence and absence of DEX. Columns represent the mean ± SEM. Differences between data sets were analyzed using one-way analysis of variance (ANOVA) followed by Tukey’s post-hoc test. **p = 0.0067. **(B)** Inhibition of proliferation of PHA-stimulated Cb-STs (grey column; n = 2 in triplicates) and penta-STs (black column; n = 2 in triplicates) upon culture with DEX. Inhibition is expressed relative to untreated (NO DEX) counterparts. Differences between data sets were analyzed using 2-tailed Student’s t-test. **p = 0.001. **(C, D)** Inhibition of IFN-γ **(C)** and TNF-α **(D)** secretion of Cb-STs (grey columns; n = 5) and their unedited counterparts (penta-STs; black columns; n = 5) upon antigen stimulation and in the presence of dexamethasone (DEX). Inhibition is expressed relative to untreated (NO DEX) counterparts. Columns represent the mean ± SEM. Differences between data sets were analyzed using 2-tailed Student’s t-test. *p < 0.05; **p < 0.01. **(E)** Cytotoxic activity of Cb-STs (grey line; n = 5 in duplicates or triplicates) and penta-STs (black line; n = 5 in duplicates or triplicates) against autologous, peptide-pulsed PHA blasts in the presence (dotted lines) or absence (solid lines) of DEX. Differences between data sets were analyzed using two-way ANOVA, followed by Dunnett’s post-hoc test. *p <= 0.011; ***p < 0.0001. **(F)** Inhibition of the cytotoxic activity of Cb-STs (n = 5 in duplicates or triplicates) and penta-STs (n = 5 in duplicates or triplicates) in the presence of DEX. Columns represent the mean ± SEM. Differences between data sets were analyzed using 2-tailed Student’s t-test. **p ≤ 0.0006. **(G)** Antifungal activity of Cb-STs (grey columns; n = 2 in triplicates) and penta-STs (black columns; n = 2 in triplicates) in the presence and absence of DEX. Columns represent the mean ± SEM. Differences between data sets were analyzed using ANOVA followed by Dunnett’s post-hoc test. ****p < 0.0001. Cb-STs, “Cerberus” T cells; Penta-STs, pentavalent-specific T cells; IFN-γ, interferon-γ; AdV, adenovirus; CMV, cytomegalovirus; EBV, Epstein Barr virus; BKV, BK virus; AF, *Aspergillus fumigatus*; TNF-α, tumor necrosis factor-α; PHA, Phytohaemagglutinin.

When non-edited penta-STs were tested in respect of their proliferation upon re-stimulation with PHA in the presence of escalating doses of DEX or Methylprednisolone, there was a considerable rate of inhibition of their proliferation, most prominent with the highest doses tested (10^-4^ M and 10^-3^ M, respectively) (**Figure S5**). We therefore considered more challenging to evaluate the resistance of Cb-STs against the high dose of 10^-4^ M DEX resulting in more than 50% inhibition of proliferation and to select DEX over Methylprednisolone due to its known increased potency at the same dose levels (42) (also confirmed in **Figure S5**). Indeed, Cb-STs provided functional resistance in the presence of high DEX dose, retaining their ability to proliferate upon re-stimulation with PHA, in contrast to the significant inhibition of penta-STs (**Figures 4A, B**). Furthermore, the functionally efficient, GR knock-out in Cb-STs was confirmed by the Cb-STs’ ability, in the presence of DEX, to secrete IFN-γ and TNF-α upon antigen stimulation, at levels similar to the no-DEX condition. In contrast, in the presence of DEX, stimulated penta-STs cytokine secretion was inhibited by 11 to 100% (**Figures 4C, D**). More importantly, the cytotoxic activity of Cb-STs against autologous AdV-pulsed PHA blasts and across different effector:target ratios was minimally impaired by DEX, in contrast to penta-STs (**Figures 4E, F**). Similarly, in the presence of DEX, Cb-STs exerted strong hyphal damage, assessed by the XTT assay whereas penta-STs ability to lyse AF hyphae was strongly inhibited by DEX (**Figure 4G**).

### Safety Profile of Cb-STs

**Figure 5:**
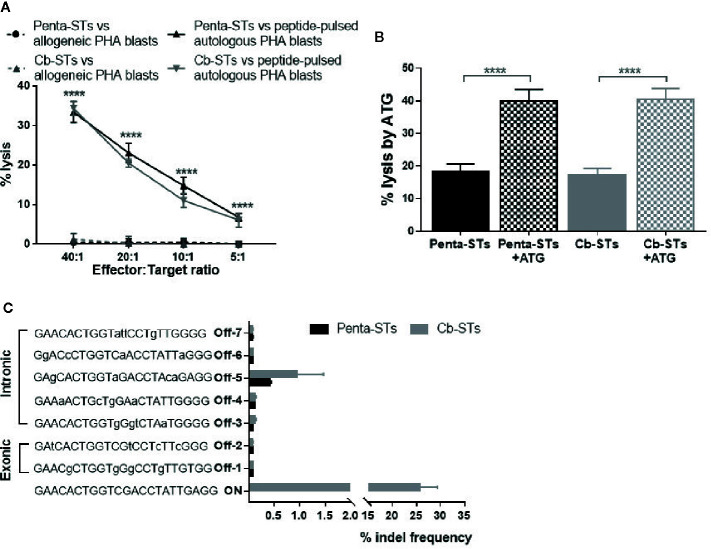
Safety profile of Cb-STs. **(A)** No alloreactivity of Cb-STs (dotted grey line, n = 4 in duplicates) against allogeneic unpulsed PHA-blasts (allogeneic targets, n = 1–3), similar to penta-STs (dotted black line, n = 5 cell products tested against allogeneic targets, n = 1–3), in contrast to strong lysis against autologous, peptide-pulsed PHA blasts (Cb-STs: grey line, n = 5 in duplicates or triplicates; penta-STs; black line, n = 5 in duplicates or triplicates). Differences between data sets were analyzed using one-way analysis of variance (ANOVA) followed by Tukey’s post-hoc test. ****p < 0.0001. **(B)** Cb-STs (grey columns; n = 5 in triplicates) remain susceptible to ATG lysis, similar to Penta-STs (black columns; n = 5 in triplicates). Columns represent the mean ± SEM. Differences between data sets were analyzed using one-way analysis of variance (ANOVA) followed by Tukey’s post-hoc test. ****p < 0.0001. **(C)** Off-target site analysis from in silico prediction using next-generation sequencing (n = 4 Cb-STs and n = 4 penta-STs). Mismatches with the DNA targeted by sgRNA are shown in small letters.

Cb-STs and their parental, non-edited penta-STs were generated by adaptation of a protocol previously used for the production of multivirus-specific T cells, which have been proved clinically safe without inducing alloreactivity (20, 22). To confirm lack of alloreactivity, Cb-STs were tested by cytotoxicity assay against unpulsed, allogeneic- versus autologous-PHA blasts and compared to the respective alloreactivity of their non-edited counterparts. Cb-STs or penta-STs cocultured with allogeneic unpulsed PHA blasts were not cytolytic (**Figure 5A**) whereas exhibited strong cytotoxic potential against autologous, peptide-pulsed PHA blasts, thus underscoring the lack of alloreactivity in combination with specific target recognition and elimination.

To address the reasonable, albeit unlikely, safety concerns of development of steroid-resistant GvHD, we tested Cb-STs susceptibility to another common immunosuppressant, ATG, as an alternative rescue treatment, if unexpected, Cb-STs-derived GvHD occurs. Importantly, Cb-STs and penta-STs were equally lysed by ATG (**Figure 5B**).

To identify potential off-target effects of the selected gRNA in Cb-STs derived from four donors, we employed a previously published algorithm (36) which allows variations in PAM sequences recognized by Cas9. Importantly, no loci with 1, 2, or 3 base mismatches were generated from this analysis. From the 25 candidate off-target sequences with four base mismatches, we analyzed seven exonic and intronic loci by deep sequencing (**Table S2**) (**Figure 5C**). Cb-STs edited with CRISPR/Cas9 had a low indel frequency at the seven potential off-target sites, similar to the ones observed in the unedited penta-STs deriving from the same donors. The high on-target editing and increased specificity with minimal off-target cutting, strongly supports the clinical translation of Cb-STs.

## Discussion

Adoptive immunotherapy with VSTs has been safely and successfully applied over the last 2 decades in multiple centers (12, 44–45). However, patients receiving high dose steroids and who are by definition, the most vulnerable to infections, are practically devoid of the benefits of AI. Intense immunosuppression drastically impairs optimal T-cell function corresponding to a lack of VST expansion *in vivo* (46, 47) while reasonably, constitutes an exclusion criterion for administering AI.

We here demonstrate the rapid production of a composite T-cell product, “Cerberus” T cells, simultaneously targeting the most common pathogens while preserving functionality within the hostile microenvironment generated by high-dose steroids. This “all-in-one” product will allow transplanted patients with opportunistic infections to enjoy the benefits of AI, regardless of the intensity of immunosuppression.

**Figure 6:**
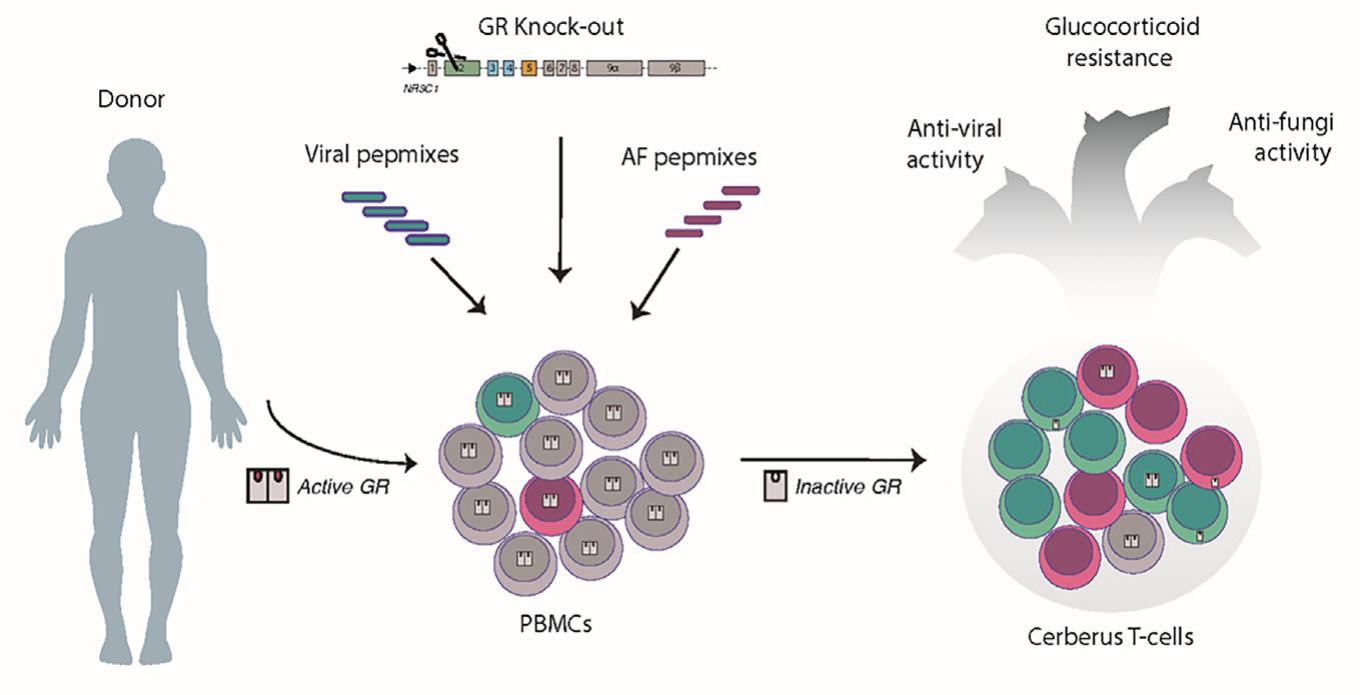
Conceptual paradigm of a “five-in-one” T cell product generation. In unedited PBMCs, once GR binds to its ligand (glucocorticoids), the GR complex enters the nucleus, and induces genomic effects. Pulsing of PBMCs with viral and AF pepmixes favors the enrichment of virus- and AF-specific T cells (green cells and pink cells, respectively). CRISPR/Cas9-ribonucleoprotein delivery results in complete disruption or partial inactivation of the GR and cytoplasmic retention of the unliganded GR. By combining peptide pulsing and CRISPR/Cas9 editing, we generate a single T-cell product, called “Cerberus” T cells, having the triple capacity to target viruses, kill fungi and remain untouchable by steroids, due to the disruption of the GR.

Menger et al. provided the first proof of concept for the development of streptamer-selected CMV-specific T cells with transcription activator-like effector nucleases (TALEN)-mediated resistance to glucocorticoids (48). Towards overcoming the hurdles of HLA-restriction associated with streptamer selection and the complexity of TALEN platform, two groups very recently reported the generation of steroid-resistant, virus-specific T cells by using the CRISPR/Cas9 platform for editing and either the IFN-γ-capture (49) or the expansion strategy for VSTs production (50). We here, broadened the targeted pathogen repertoire by generating pentavalent Ag-specific T cells, named “Cerberus”, after the three-headed, guardian dog of the underworld due to their triple functionality of protecting against 4 viruses, at least 1 fungus (AF) and being resistant to steroids by CRISPR/CAS9-mediated disruption of the GR (**Figure 6**).

In order for cell therapy to become a viable and both user- and recipient-friendly therapeutic strategy, the manufacturing process should be rapid and simple and the cell product be administered as one-time treatment, providing long-lasting protection against multiple, common pathogens. We here propose a rapid and simplified manufacturing process for both the generation of multipathogen-specific T cells and the genetic inactivation of the GR, to ultimately produce Cb-STs. By using ex vivo expansion of VSTs under a previously developed protocol (20, 22), the need for leukapheresis as a starting manufacturing material or/and high frequency of circulating VSTs as when direct selection techniques are applied, was overcome. We also extended the repertoire of targeting to generate pentavalent T-cell products, including, in addition to viruses, the fungus AF, successfully addressing the challenge to effectively expand the less frequent in blood, as compared to VSTs, aspergillus-specific T cells (51). Importantly, as we have also shown previously (34), stimulation of AF-STs or multi-pathogen–STs with other fungi (Candida, Fusarium) results in expansion of cross-strain-immunity and a broader than reasonably anticipated, killing repertoire and overall fungal protection.

Moreover, the use of CRISPR/Cas9 editing platform has a number of advantages over other designer nucleases in terms of practicality and efficiency, allowing for efficient and labor-saving targeting of genome sites. By CRISPR/Cas9-mediated GR editing of penta-STs, and despite the known electroporation-induced impairment of T cell viability and functionality, we reached from a small amount of peripheral blood, approximately 2x10^8^ cells, a dose one log higher than what is usually required for clinical application.

Importantly, the editing process did not alter the cells’ phenotypic and functional characteristics and Cb-STs preserved the expansion ability, immunophenotype and specificity of penta-STs. In particular, Cb-STs consisted of both helper and cytotoxic subpopulations and expressed memory markers known to allow long-term persistence *in vivo* (52). GR knock-out not only did not devoid Cb-STs from their ability to expand and function against all targeted viral and fungal pathogens, but importantly, allowed for their proper functionality even in the presence of glucocorticoids.

The clinical use of VSTs in the transplant setting has a proven long record of increased safety. In contrast to CAR T-cells (53), VSTs have been associated with minimal occurrence of Cytokine Release Syndrome (CRS) (54, 55). In addition, VSTs-associated GvHD grade≥2 was infrequently observed in the allo-HCT setting (16, 18, 20, 56), even when VSTs were derived from third party donors (22, 57), while no toxicity or evidence of rejection was observed in the SOT setting (12). Likewise, we expect that our Cb-STs, produced on the basis of a clinically-applied VST-protocol (20, 22) will incur minimal risk of GvHD or CRS in allo-HCT patients. Further supporting this, in a proof of concept study, we have very recently shown that multi-pathogen-specific T–cells did not incur alloreactivity while effectively controlled EBV-associated, Diffuse Large B-cell Lymphoma in a humanized mouse model [(41), in press]. In the unlikely case however, of induced alloreactivity that could be steroid-resistant, we show that Cb-STs remain susceptible to complement-mediated lysis by ATG, similarly to the unedited penta-STs, allowing the *in vivo* elimination on demand.

In order to mitigate safety concerns arising from unwanted genome modification (58), we screened 8 individual sgRNAs targeting various exons of the GR gene. Among the 6 effective sgRNAs, we selected the e2b sgRNA, as having the lowest off-target predicted cutting and presenting in silico off-target cleavage activity only with ≥4 base pair mismatches in the PAM-distal part of the sgRNA-guiding sequence. The e2b sgRNA/Cas9 was delivered as a protein complex (RNPs) in order to “hit” the target immediately after delivery and then be rapidly broken down by endogenous proteases (58). The “hit and run”, transient expression of CRISPR/Cas9 after RNP electroporation offers advantages towards clinical translation, as not only reduces the risks of off-target gene modification but also the risks of insertional mutagenesis, by avoiding genome integration of a delivery vector. After comprehensively testing mutagenesis at 7 predicted genomic loci, we observed no meaningful off-target activity, thus strongly supporting the potential for clinical translation.

In conclusion, we are introducing a novel, rapid and GMP-compatible protocol for clinical scale generation of a single T-cell product with the capacity to target the most common viral and fungal pathogens affecting transplanted patients, while being untouchable by the most favored immunosuppressant. Cb-STs as a powerful, “five-in-one” T-cell product could considerably reduce the transplant-related mortality associated with opportunistic infections and ultimately improve the outcome of allo-HCT.

## Data Availability Statement

The original contributions presented in the study are included in the article/**Supplementary Material**, while the NGS data are deposited in the ArrayExpress repository, accession number E-MTAB-9968. Further inquiries can be directed to the corresponding authors.

## Ethics Statement

The study was approved by the Institutional Review Board of the George Papanikolaou hospital. Under signed informed consent, peripheral blood from healthy volunteers was obtained for the generation of antigen-specific T cells.

## Author Contributions

Conceptualization, AP and EY. Methodology, KK, P-GP, AG, MA, SL, AK, CP, T-AV, GG, and AP. Investigation, KK, P-GP, AG, MA, LS, AK, CP, AS, AM, AC, NP, and EY. Writing—original draft, NP, EY, and AP. Writing—review and editing, NP, EY, and AP. Funding acquisition, AP, AA, and EY. Resources, AA and EY. Supervision, AA and EY. All authors contributed to the article and approved the submitted version.

## Funding

Funding for this project was provided by an advanced EHA (European Hematology Association) Research Grant award.

## Conflict of Interest

The authors declare that the research was conducted in the absence of any commercial or financial relationships that could be construed as a potential conflict of interest.
