# Supplementary material for: “Cerberus” T Cells: A Glucocorticoid-Resistant, Multi-Pathogen Specific T Cell Product to Fight Infections in Severely Immunocompromised Patients
Source: Front Immunol. 2021 Jan 18;11:608701. doi: 10.3389/fimmu.2020.608701 (PMC7848034; doi:10.3389/fimmu.2020.608701)
Supplement: Supplementary file 1 [file DataSheet_1.docx]

**Supplementary tables**

**Table S1**

| **Table S1. Guide RNAs used for gene knockout** | | | | |
| --- | --- | --- | --- | --- |
| **Name** | **5’ gRNA sequences** | **Target site domains** | **On-target specificity score** | **Off-target specificity score** |
| **e1** | CGAGCGAGCGGGACCGAGCG | Exon 1 | 30 | 77 |
| **e2a** | ACCAGGAGTTAATGATTCTT | Exon 2 | 66 | 39 |
| **e2b** | GAACACTGGTCGACCTATTG | Exon 2 | 72 | 90 |
| **e2c** | GGCCAGACTGGCACCAACGG | Exon 2 | 67 | 75 |
| **e2d** | TTAGAAAAAACTGTTCGACC | Exon 2 | 39 | 59 |
| **e4** | CCGCTATCGAAAATGTCTTC | Exon 4 | 34 | 84 |
| **e5a** | GTGAGTTGTGGTAACGTTGC | Exon 5 | 61 | 78 |
| **e5b** | ATGACTACGCTCAACATGTT | Exon 5 | 58 | 79 |

**Table S2**

| **Table S2. List of ON/OFF-target sequences and primers used for Miseq analysis** | | | | | |
| --- | --- | --- | --- | --- | --- |
| **Name** | **Chromosome** | **Mismatches** | **Targeted position** | **Forward primer** | **Reverse primer** |
| **On** | chr5 | 0 |  | TCGTCGGCAGCGTCAGATGTGTATAAGAGACAGT  GCTGAACTCTTGGGGTTCT | GTCTCGTGGGCTCGGAGATGTGTATAAGAG  ACAGACTGGCTGTCGCTTCTCAAT |
| **Off-1** | chr15 | 4 | EXONIC | TCGTCGGCAGCGTCAGATGTGTATAAGAGACAG  CTCCTCAACCCACAGCAC | GTCTCGTGGGCTCGGAGATGTGTATAAGAG  ACAGTGTACAGCTTGCCCAGGT |
| **Off-2** | chr16 | 4 | EXONIC | TCGTCGGCAGCGTCAGATGTGTATAAGAGACAGG  CTCAACCACTTGCCAAACT | GTCTCGTGGGCTCGGAGATGTGTATAAGAG  ACAGCCCCGATACACGGTGTCC |
| **Off-3** | chr7 | 4 | intronic | TCGTCGGCAGCGTCAGATGTGTATAAGAGACAGC  ATGCATTGAGTGGAAACCA | GTCTCGTGGGCTCGGAGATGTGTATAAGAG  ACAGCGCCATGTTATGAAGTCAGC |
| **Off-4** | chr8 | 4 | intronic | TCGTCGGCAGCGTCAGATGTGTATAAGAGACAGG  CCCAGGGATTACGTAAAGG | GTCTCGTGGGCTCGGAGATGTGTATAAGAG  ACAGTAGAGGTGGCATTTTATGCTGA |
| **Off-5** | chr10 | 4 | intronic | TCGTCGGCAGCGTCAGATGTGTATAAGAGACAG  TCCATGGTCACAGCCATTTA | GTCTCGTGGGCTCGGAGATGTGTATAAGAG  ACAGGACTTGCCGCTTGATCTGTT |
| **Off-6** | chr11 | 4 | intronic | TCGTCGGCAGCGTCAGATGTGTATAAGAGACAG  AAGGGGGTTATGACATCTGTTT | GTCTCGTGGGCTCGGAGATGTGTATAAGAG  ACAGTGCCAGAGATCCAGAGATGA |
| **Off-7** | chr16 | 4 | intronic | TCGTCGGCAGCGTCAGATGTGTATAAGAGACAG  CAACCCTCAGGAGACCAAAA | GTCTCGTGGGCTCGGAGATGTGTATAAGAG  ACAGCCAACCTTAGCCAGAGCCTA |

**Table S3**

| **Table S3. Vector copy number** | |
| --- | --- |
| **gRNA** | **VCN** |
| **e1** | 0.8 |
| **e2a** | 0.8 |
| **e2b** | 1.0 |
| **e2c** | 0.7 |
| **e2d** | 0.5 |
| **e4** | 0.9 |
| **e5a** | 0.6 |
| **e5b** | 0.6 |

**Supplementary figures**

**Figure S1**

**
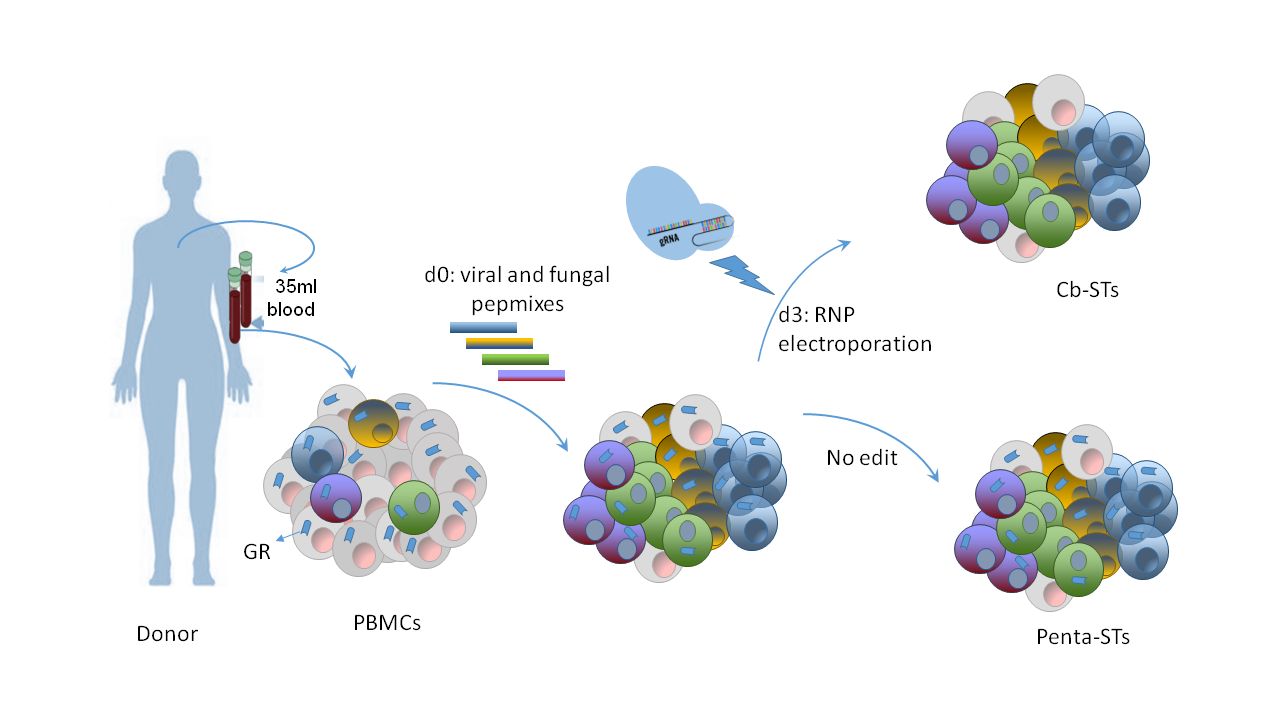
**

**Figure S1. Procedural overview of the platform for generating Cb-STs.** Peripheral blood mononuclear cells (PBMCs) are isolated from 35ml of donor blood, pulsed with a mastermix of viral and AF peptides and cultured for 3 days. At day 3, Cas9 RNPs are formed *in vitro* by incubating Cas9 protein with the newly formed sgRNA and electroporated into 3x10^7^ of activated T cells, subsequently expanded in culture for 7 additional days. The remaining unedited penta-STs served as control group. Cb-STs: Cerberus-T cells; AF: Aspergillus fumigatus; RNPs: ribonucleoproteins; penta-STs: pentavalent-specific T cells.

**Figure S2**

**
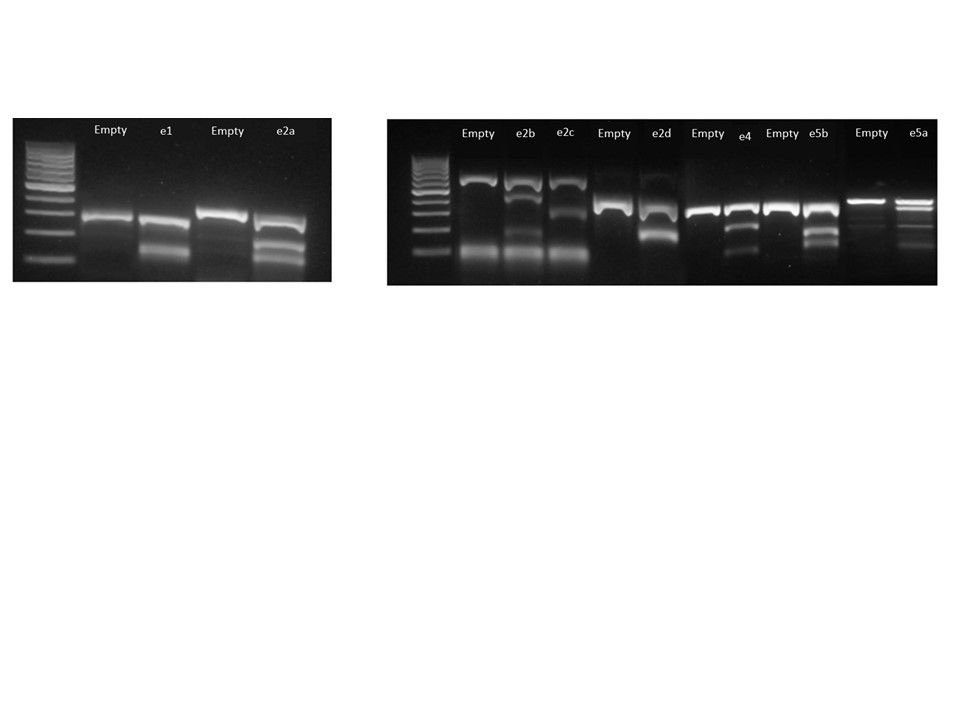
**

**Figure S2.** Representative gel images of T7E1-treated PCR products amplified from the target sites of negative controls (empty-vector transduced T2 cells) and edited T2 cells.

**Figure S3**

**
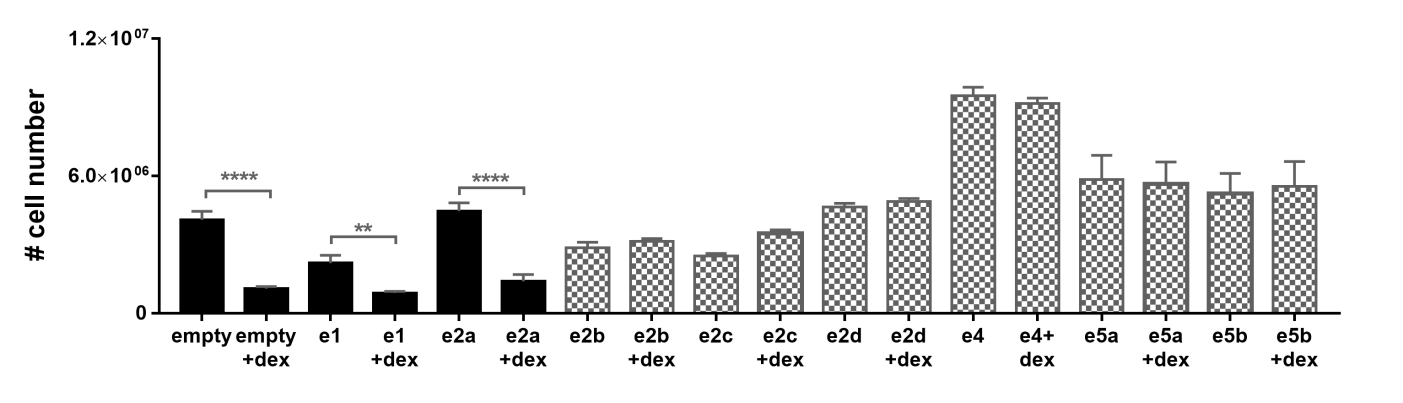
**

**Figure S3.** Proliferation of edited and unedited (empty) T2 cells in the presence and absence of dexamethasone, showing resistance to dexamethasone in T2 cells edited with 6 (filled grey) out of 8 gRNAs. Columns represent the mean±SEM. Differences between Dex-treated cells and their untreated counterparts were analyzed using 2-tailed Student’s t-test. **p=0.002 Dex-treated vs untreated cells; ****p<0.0001 Dex-treated vs untreated cells.

**Figure S4**

**
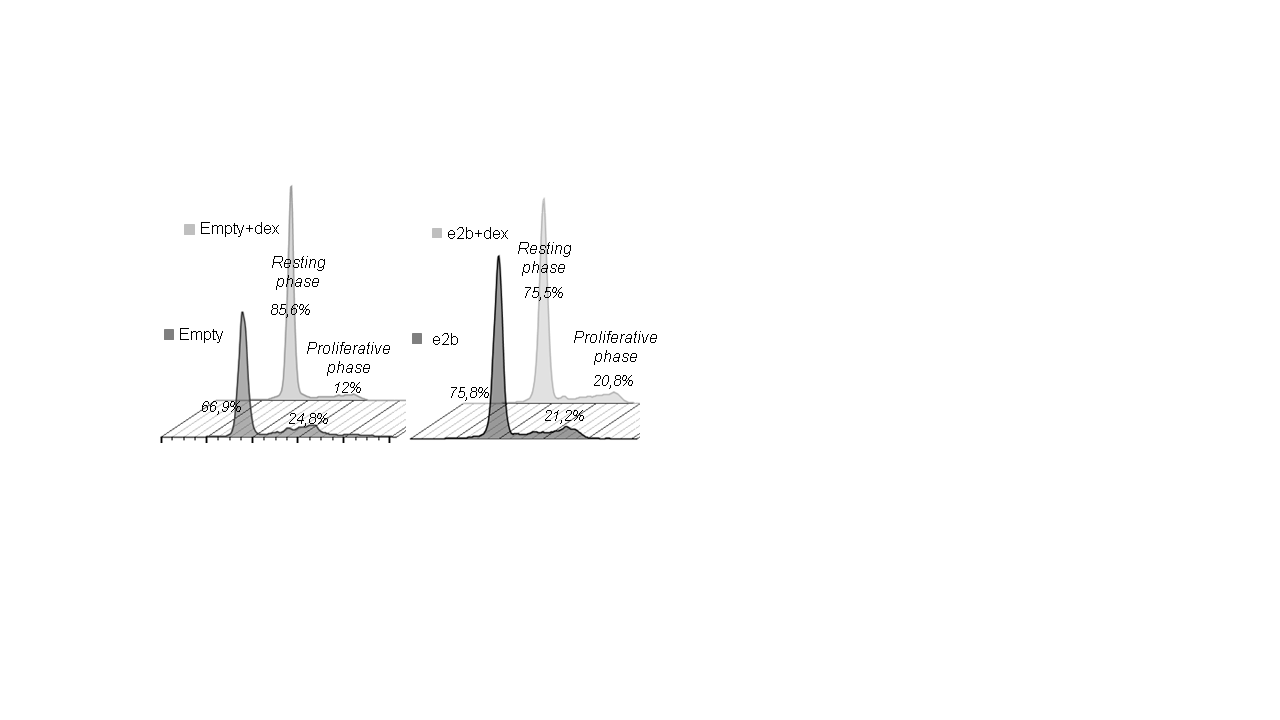
**

**Figure S4.** Representative flow cytometry histograms of propidium iodide fluorescence distributions in edited (e2B) and unedited (empty) T2 cells in the presence (light grey) and absence of DEX (dark grey). The cells were quantified by their relative distribution in the GO/G1 zone of the DNA fluorescence histograms (resting phase), and S/G2/M (proliferative phase).

**Figure S5**

**
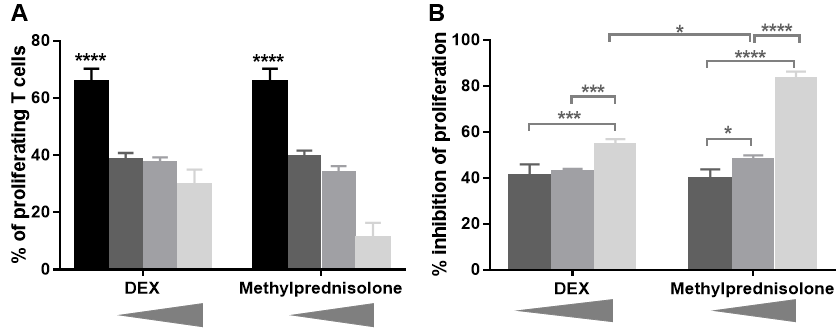
**

**Figure S5. A)** Proliferation of PHA-stimulated penta-STs in the presence and absence of different doses of DEX (0.5x10^-5^ M or 10^-5^ M or 10^-4^ M) or methylprednisolone (10^-5^ M or 10^-4^ M or 10^-3^ M; n=2 in triplicates). Columns represent the mean±SEM. Untreated PHA-pulsed penta-STs (black columns); DEX or methylprednisolone-treated PHA-pulsed penta-STs (grey columns). Differences between data sets were analyzed using one-way analysis of variance (ANOVA) followed by Tukey’s post-hoc test. ****p<0.0001. **B)** Inhibition of proliferation of PHA-stimulated penta-STs upon culture with different doses of DEX (0.5x10^-5^ M or 10^-5^ M or 10^-4^ M) or methylprednisolone (10^-5^ M or 10^-4^ M or 10^-3^ M; n=2 in triplicates). Inhibition is expressed relative to untreated (NO DEX or methylprednisolone) counterparts. Differences between data sets for DEX and methylprednisolone conditions were analyzed using one-way analysis of variance (ANOVA) followed by Tukey’s post-hoc test. *p=0.04; ***p≤0.0008; ****p<0.0001. Differences between DEX-treated vs methylprednisolone-treated cells at 10^-4^ M were analyzed using 2-tailed Student’s t-test. *p=0.003.
